# Reliable Task Management Based on a Smart Contract for Runtime Verification of Sensing and Actuating Tasks in IoT Environments

**DOI:** 10.3390/s20041207

**Published:** 2020-02-22

**Authors:** Lei Hang, Do-Hyeun Kim

**Affiliations:** Department of Computer Engineering, Jeju National University, Jeju 63243, Korea; hanglei@jejunu.ac.kr

**Keywords:** Internet of Things, task management, blockchain, smart contract, runtime verification

## Abstract

With the gradual popularization of Internet-of-Things (IoT) applications and the development of wireless networking technologies, the use of heterogeneous devices and runtime verification of task fulfillment with different constraints are required in real-world IoT scenarios. As far as IoT systems are concerned, most of them are built on centralized architectures, which reveal various assailable points in data security and privacy threats. Hence, this paper aims to investigate these issues by delegating the responsibility of a verification monitor from a centralized architecture to a decentralized manner using blockchain technology. We present a smart contract-based task management scheme to provide runtime verification of device behaviors and allows trustworthy access control to these devices. The business logic of the proposed system is specified by the smart contract, which automates all time-consuming processes cryptographically and correctly. The usability of the proposed solution is further demonstrated by implementing a prototype application in which the Hyperledger Fabric is utilized to implement the business logic for runtime verification and access control with one desktop and one Raspberry Pi. A comprehensive evaluation experiment is conducted, and the results indicate the effectiveness and efficiency of the proposed system.

## 1. Introduction

A typical IoT system is usually a combined system with computation and communication capabilities and provides physical environments, in which numerous embedded devices are connected to provide services to end-users [1]. Thus far, IoT technology has been widely adopted in all aspects of our daily lives, such as intelligent transportation, smart homes, and manufacturing industries, using machine-to-machine communication [2,3]. It is estimated that the number of connected IoT devices will increase from 15.4 billion to 75.44 billion worldwide by 2025, a five-fold increase in ten years [4]. 

Current IoT systems are generally built on a highly centralized architecture, where cloud-based servers provide the capabilities of data analysis and processing over the Internet. Despite the data management abilities and magnificent elastic computation provided by this solution, the system error and software security flaws sharply increase as IoT systems become increasingly complicated and broad. One of the disadvantages of this solution is that it may introduce a single point of failure that can compromise the availability of networks or servers and is not suitable for applications that require high performance [5]. While the network scale of IoT systems is growing, a large amount of sensing data would significantly increase the processing effort, thus increasing the burden of the centralized verification monitor and reducing the stability of the whole system [6]. 

Solving the security flaw of a single point of failure and ensuring the stability and reliability of conventional IoT systems can be achieved by using decentralized technology, called a blockchain [7]. From a conceptual level, a blockchain is a distributed ledger consisting of a chain of blocks that are replicated by the nodes of a distributed network [8]. Every block of the ledger is logically linked to the previous block by storing the hash of the parent. The hash of a prior block is designed to be tamper-proof; thus, the user can easily use the verification process to detect the discrepancy since a single bit change in a given block would alter all of the previous transaction logs in the block. As blockchain technology becomes more mature, use cases and applications outside of financial services are gradually being explored in various business scenarios, such as healthcare [9,10,11], IoT [12,13,14], smart grid [15,16,17], and shared economy applications [18,19,20]. 

This paper introduces a novel approach to migrate the verification monitor from a centralized architecture into a decentralized way by using blockchain technology. The smart contract provides the ability to monitor whether the running status of a device behaves as expected, and then enforce the rule to correct the unusual events. The smart contract is deployed on a secure, permissioned network without the use of cryptocurrencies, thus diminishing the threat of malicious program infection via the smart contract and ensuring rapid and stable transaction processing. The services provided by the blockchain network are exposed as REST APIs through which the external applications and IoT devices can interact with the blockchain network without any modification. 

Additionally, we apply a separate data storage solution by deploying the non-SQL database, namely Couch DB, residing on each network node to serve as the file storage of the whole network. To demonstrate the feasibility of the proposed solution, a prototype application is developed in which the Hyperledger Fabric [21] framework is utilized to implement the smart contract for task management in an IoT system with one desktop and one Raspberry Pi.

Main contributions of this paper can be summed up as follows:

1. This paper introduces a task management scheme specified to IoT systems in a distributed and trusted manner. The smart contract specified the application logic of the IoT system, including device registration, task generation, task allocation, and task verification. The proposed scheme guarantees the IoT data integrity and provides a transparent record of the whole system operation history.

2. A separate data storage solution is applied by deploying the Couch DB on each network node to serve as the file storage for the sheer volume of IoT data. This approach supports productive queries that are flexible against large indexed data stores and provide various APIs to manipulate the data.

3. A RESTful-based server is proposed to integrate the external applications and IoT devices with the blockchain by exposing services provided by the smart contract as web APIs. This strategy provides an easy way to interact with the blockchain, and it is flexible and scalable to meet the demand for different application requirements.

The remainder of this paper is structured as follows. Section 2 discusses some existing studies on runtime verification approaches and the combination of blockchain with IoT systems. Section 3 describes the proposed system architecture. Section 4 elaborates on the implementation of the prototype application in depth. Section 5 evaluates the transaction processing capability of different network scales. Section 6 discusses some potential applications and challenges towards the objective of this work. Finally, Section 7 summaries the research work of the paper and indicates the future research direction.

## 2. Related Works

A typical IoT environment is composed of numerous connected, configurable, and smart devices with limitations in memory and computing power. These limitations can result in service failure on the device that can lead to a complete application failure. Hence, a runtime verification approach is necessary to verify if the observed device running behaviors match the predefined expected action. 

There exist some studies on runtime verification of IoT applications. For example, the authors in [22] present a runtime monitoring approach for IoT systems by utilizing event relations. Additionally, they propose the use of Complex-Event Processing (CEP) to verify device running behaviors through defined event algebra. Another similar work on runtime verification for IoT systems [23] exploits the interactions via message sequence charts (MSC) to specify message exchange in terms of events. They describe event constraints by using a new event calculus and apply CEP to detect service failures by monitoring the runtime event occurrences if the condition is triggered. The authors in [24] present a novel runtime verification and related prototype implementations to demonstrate the usability and efficiency of the proposed solution. They apply a rule-based pattern to detect the events generated by devices in real-time. The processing overhead of the verification mechanism is low, according to the results. The authors in [25] present a novel solution framework that is first of its kind in proposing the democratization of runtime verification based on a model-based testing approach. Runtime events from devices are collected and further transferred to a cloud CEP engine for analysis. The proposed solution is demonstrated by implementing an ambient assisted living (AAL) system that consists of healthcare sensors and actuators. A predictive runtime verification framework is proposed to predict the runtime failures of IoT devices before a real failure happens [26]. A control flow graph-based prediction is implemented on top of the proposed framework, where runtime information is used to do speculative execution and improve the prediction accuracy. 

As far as the combination of blockchain and IoT systems is concerned, the authors provide an overview of the combination of the blockchain with IoT and highlight their benefits, challenges, and future directions [27]. This paper indicates that migrating the centralized IoT system into a decentralized path is the right decision as decentralized features of the blockchain in computation and management processes can be a powerful technology to solve many IoT security issues. The authors propose a lightweight blockchain-based architecture to eliminate the overheads of conventional blockchains [28]. This system is based on multiple chains, including a private immutable ledger used to optimize energy consumption from IoT devices, and a public ledger used to ensure end-to-end security and privacy. The authors in [29] present a fully-distributed access control system for IoT based on a blockchain. A single Ethereum smart contract is used to simplify the process in the blockchain network and reduce the communication overhead between IoT nodes. The proposed architecture is backed by a case study implementation in real IoT scenarios. A blockchain connected gateway is presented in [30] to maintain user privacy preferences in the blockchain network. This approach eliminates the possibility of privacy leakage as the gateway protects users’ sensitive data from being accessed without their consent. The authors in [31] propose a smart contract-based framework, which consists of multiple contracts specified to different functions, including access control, judgment, and registering. The usability of the proposed framework is evaluated by a case study in an IoT system with one desktop, one laptop, and two Raspberry Pi. The authors in [32] first introduced a blockchain-based approach for edge computing systems, where IoT devices and end-users can access physical resources or consume services from an edge service provider. An edge computing enabled blockchain prototype is implemented as the proof of concept, and the results demonstrate the usability and efficiency of the proposed solution. A similar approach is presented in [33] to ensure secure authentication and data sharing for different IoT platforms. Unlike the Proof of Work (PoW) consensus algorithm used in [32], this work utilizes an optimized Practical Byzantine Fault Tolerance (PBFT) algorithm to improve the transaction throughput. The smart contract is used to provide edge related services, including name resolution and edge authentication services. Additionally, the authors propose a caching-based approach to improve the hit ratio of the designed system, and the experiment results indicate that the proposed solution outperforms existing edge computing systems by 8–14% in terms of hit ratio. The authors in [34] overview some of the current works related to the integration between the edge computing system and blockchain. They also discuss some research issues and challenges, covering self-organization, system scalability, function integration and, most import of all, security issues.

As mentioned, these works either provides runtime verification in a centralized manner or focus on the combination of blockchain with IoT devices. To the best of knowledge of the authors, this paper makes the first attempt to implement a decentralized runtime verification for IoT systems using blockchain technology. The current IoT system can tremendously benefit from the decentralized runtime verification approach in reducing the burden of verification overload on the cloud-based server. 

## 3. Proposed System Architecture

### 3.1. System Overview

**Figure 1:**
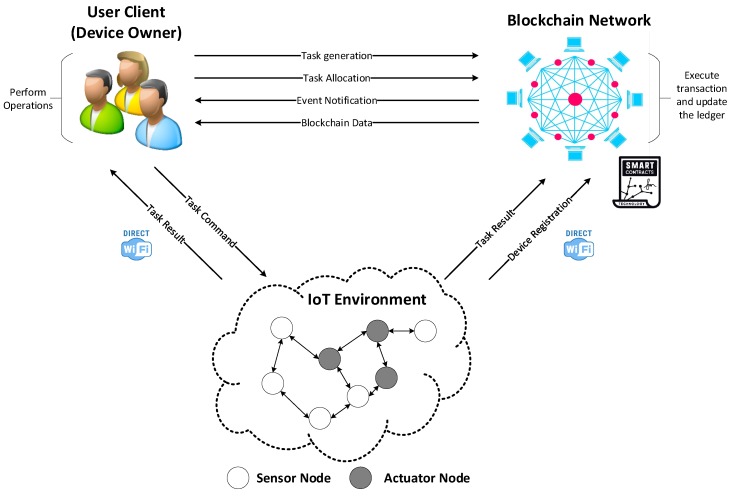
Conceptual architecture of the proposed system.

Figure 1 describes the proposed system conceptual architecture, which is comprised of three main components: the IoT environment, the blockchain network, and the user client. The IoT environment is formed by various sensor nodes used for environmental monitoring and actuator nodes for environment adjusting. These sensor and actuator nodes exchange information through a shared network and these sub-networks form up to construct a global IoT environment. The communication between the IoT environment, blockchain network, and user client happens over wireless communication protocols, such as Wi-Fi. The user client can perform various operations to both the blockchain network and IoT environment, such as create a task in the blockchain or assign the task to a specific device. These operations are specific transactions defined by the smart contract in the blockchain network. The blockchain network contains the smart contract and data storage to write a block of the transaction to the ledger. The smart contract executes transactions against the current state data, for instance, adding a new key-value or updating the key-value for the specific asset. 

### 3.2. Main Functions of the Proposed System

**Figure 2:**
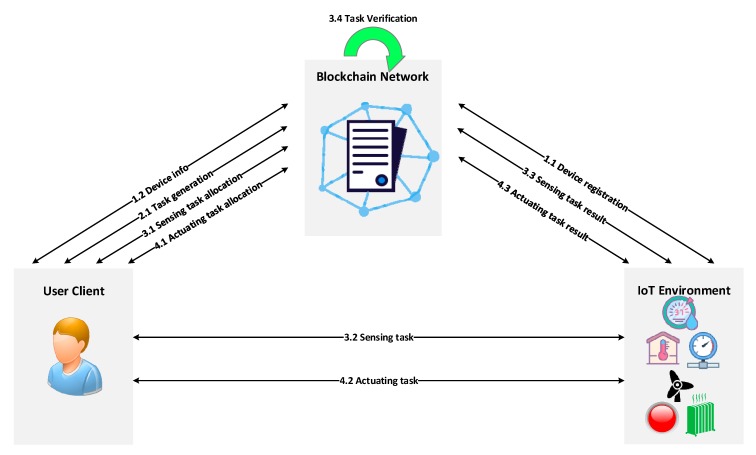
Service scenarios of the proposed system.

With the help of a smart contract, the proposed system can provide numerous functions, which are depicted in Figure 2. These functions mainly include device registration, task generation, task allocation (sensing/actuating task), and task verification. The process of each function is explained as follows.

1. Device Registration Method: The IoT device interacts with the blockchain network to register its profile through the following steps:
  - Step 1: The device submits a transaction proposal to the smart contract along with its profile. The nodes of the blockchain network execute the transaction proposal and append the transaction in the ledger. An event is emitted from these nodes to inform the device of whether the transaction succeeded or failed.
  - Step 2: The device owner requests the smart contract to query the device info that is stored in the ledger. The blockchain retrieves the device info from the ledger and sends the results to the device owner as a response.
2. Task Generation Method: The device owner interacts with the blockchain network to generate tasks for specified devices. The task is an operation that is directly associated with IoT resources such as sensors or actuators. As a result, the task can be further separated into two categories: sensing tasks and actuating tasks.
  - Step 1: The device owner specifies the task info, including task ID, name, device URI, threshold level, range, etc. Afterward, the device owner submits a transaction proposal to the smart contract along with the task info. The nodes of the blockchain network execute the transaction proposal and append the transaction in the ledger. An event is emitted from these nodes to inform the device of whether the transaction succeeded or failed.
3. Sensing Task Allocation Method: The device owner interacts with the blockchain network to record the sensing task that will be allocated through the following steps:
  - Step 1: The device owner submits a transaction proposal to the smart contract along with the sensing task.
  - Step 2: The nodes of the blockchain network execute the transaction proposal and append the transaction in the ledger. If the blockchain network permits the request of allocating the sensing task, an event is emitted from these nodes to inform the device owner that he/she can assign the sensing task to the sensor.
  - Step 3: The sensor acts according to the sensing task and sends a transaction proposal to the smart contract along with the task execution results. The nodes of the blockchain network execute the transaction proposal and append the transaction in the ledger. If the sensing value from the proposal triggers the predefined condition in the smart contract; for example, the sensing value exceeds the threshold level. An event is emitted from these nodes to inform the device owner that he/she can allocate the actuating task to the actuator.
4. Actuating Task Allocation Method: The device owner interacts with the blockchain network to record the actuating task that will be allocated through the following steps:
  - Step 1: The device owner submits a transaction proposal to the smart contract along with the actuating task.
  - Step 2: The nodes of the blockchain network execute the transaction proposal and append the transaction in the ledger. If the blockchain network permits the request of allocating the actuating task, an event is emitted from these nodes to inform the device owner that he/she can assign the actuating task to the actuator.
  - Step 3: The actuator acts according to the actuator task and sends a transaction proposal to the smart contract along with the task execution results. The nodes of the blockchain network execute the transaction proposal and append the transaction in the ledger. An event is emitted from these nodes to inform the device owner that the actuator status is changed.
5. Task Verification Method: The smart contract validates the interactions between blockchain and devices according to predefined rules based on the specified behavior of devices. The behavior of a device is specified with constraints, and one or more rules are required to perform the runtime verification.

### 3.3. Blockchain Network of the Proposed System

**Figure 3:**
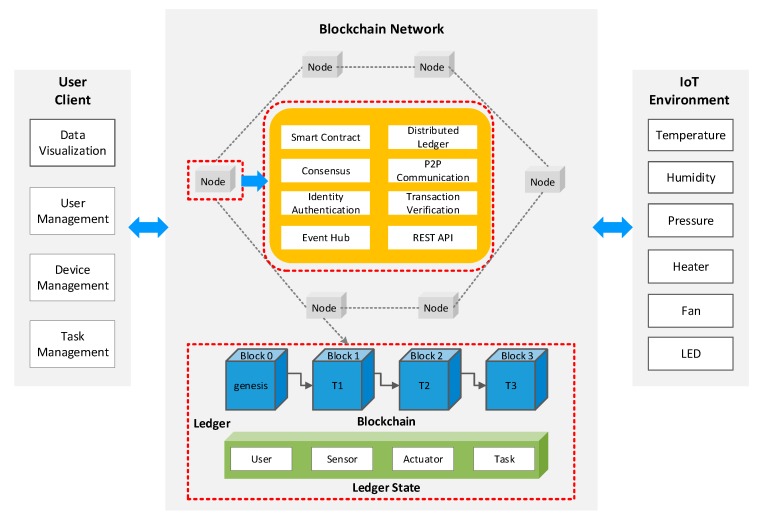
The detailed structure of the blockchain network.

The blockchain network of the proposed system is built on a permissioned network that only allows access from authorized users, which is featured in Figure 3. In a permissioned blockchain network, authorized users operate on the blockchain, and a valid block must contain a signature from a subset of users. This ensures that no invalid node could modify or insert a transaction in the blockchain. Additionally, the approved smart contract cannot be altered unless it gets signed agreement from all users featured on the operation. This solution avoids the risk of data exposure as a transaction history that records how a resource is manipulated are invisible to unauthorized users. The certificate authority (CA) is the authorized service provider, which is responsible for generating PKI-based certificates and critical material to network entities and their users. 

The blockchain network consists of multiple nodes, which acts as the host of the smart contract and distributed ledger. The ledger can be divided into two parts: blockchain and ledger state. The blockchain is a growing list of records, called blocks, which are structured as hash-linked blocks. Each block contains a hash value of the previous block, a timestamp, and transaction data (generally represented as a Merkle tree root hash). In this way, all transactions on the ledger are sequenced and cryptographically linked together. It is not possible to tamper with the ledger data unless breaking the hash links. The ledger state represents the latest values of all keys ever included in the transaction log for efficient reads and queries from smart contracts. Any changes to the ledger are reflected in all copies in minutes, or in some cases, seconds. The smart contract is a kind of code invoked by the external client applications to manage access and modifications in the ledger. All the rules and conditions are not only pre-defined by smart contracts but are also enforced by them. Digital signatures and immutable hash functions were deployed to ensure the integrity of data in the blockchain network. Moreover, it verifies the authentication of a transaction occurring within the network. The user client provides various services such as data visualization, user management, device management, and task management. The IoT environment consists of multiple sensors and actuators, such as temperature and humidity sensors, LEDs, and fans. The user client and IoT environment can invoke services specified in the smart contract to interact with the blockchain network through the REST API, which exposes the services provided by the blockchain network.

### 3.4. Runtime Verification Using the Smart Contract 

In this paper, all the business operations are handled by the smart contract, which contains the logic of the blockchain network. The logic of the smart contract is a set of rules that define how transactions will be executed and how the world state of the ledger will change. The smart contract specifies various functions that allow users to interact with the ledger. The application running on the smart contract receives the transaction from the client and invoke the associated function to perform different kinds of operations (e.g., update, query) on the ledger. The blockchain network appends the approved transaction in the block and updates the ledger state after consensus is achieved among different network entities. Finally, the blockchain network emits the transaction execution result as an event to notify the client whether the transaction is validated or not.

**Figure 4:**
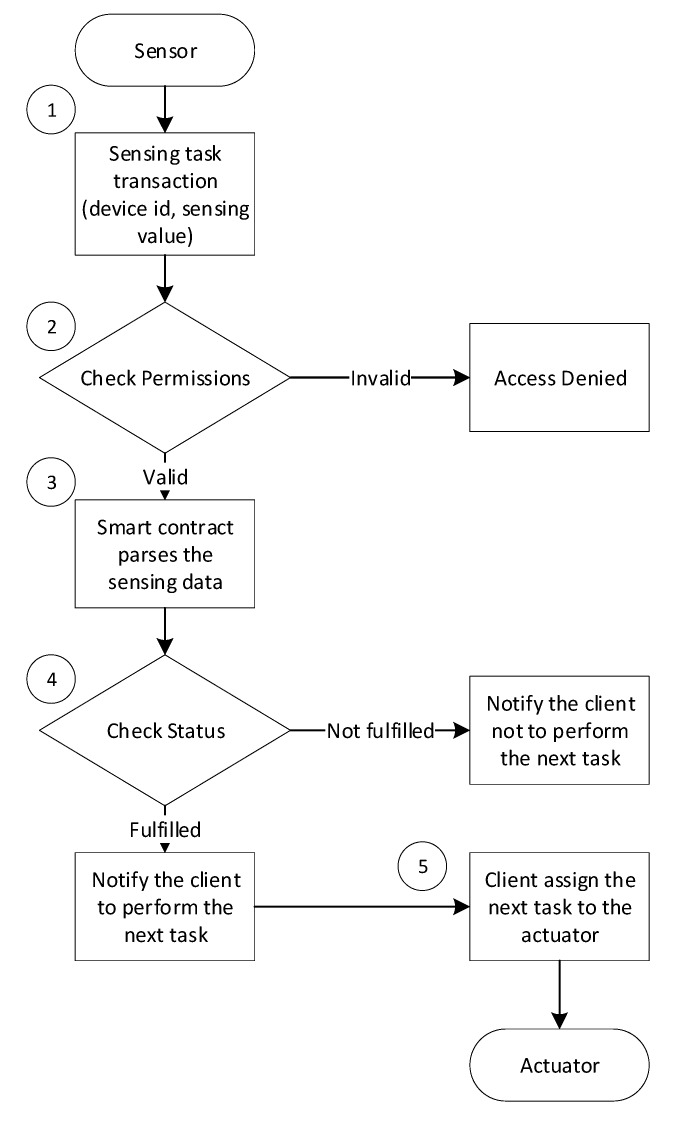
Flow chart of the execution and verification of a task.

As shown in Figure 4, the components take part in the task verification process include a sensor, smart contract, user client, and actuator. The process of the flow chart contains the following steps:

1. Step 1. The sensor invokes the smart contract and sends a sensing task transaction proposal containing the device ID and sensing value.
2. Step 2. The smart contract validates the permission of the sensor by using the device ID to check if it exists in the blockchain. The access is denied if the sensor is not registered in the network.
3. Step 3. The smart contract parses the sensing data from the transaction proposal.
4. Step 4. The sensing data parsed from the transaction proposal is used to compare with the threshold defined in the rule. If the condition is fulfilled, then the smart contract will emit an event to inform the client that it can perform the next task, and otherwise notification will be sent to inform the client that the condition is not fulfilled.
5. Step 5. The client assigns the next task to the actuator, and the actuator performs accordingly.

### 3.5. Execution Process of the User Enrolment and Device Registration 

**Figure 5:**
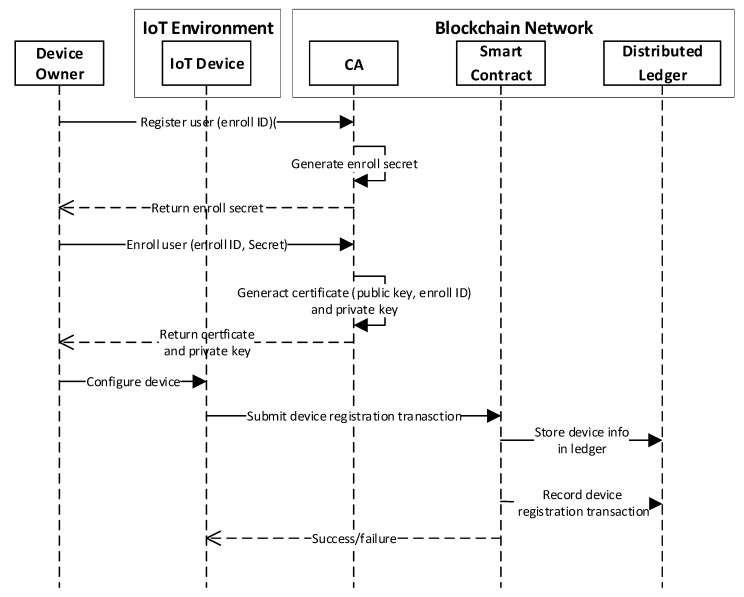
The operation process of user enrolment and device registration.

Figure 5 depicts the operation process of user enrollment and device registration. In this system, the device owner is the only participant who can perform operations on the blockchain network. As mentioned earlier, only the authorized user can access and manipulate the resources of the blockchain network. To receive the required certificate, the device owner needs to send the registration request with the enroll ID to the CA. The CA generates the enroll secret and sends it back to the device owner. The device owner makes another enrollment request to the CA using the enroll ID and secret obtained in the registration phase. The CA generates the certificate (public key, enroll ID) along with the private key and sends them back to the device owner. The device owner configures the device settings to bind the device to the obtained certificate so that the device can be authenticated by the CA and perform transactions. A device registration transaction that contains the device profile is submitted to the smart contract; in turn, the transaction is appended in the block and a new instance in the world state is generated to represent the device. Finally, an event that indicates whether the transaction is validated or not is sent back to the device.

## 4. Prototype Implementation 

### 4.1. Development Environment

**Figure 6:**
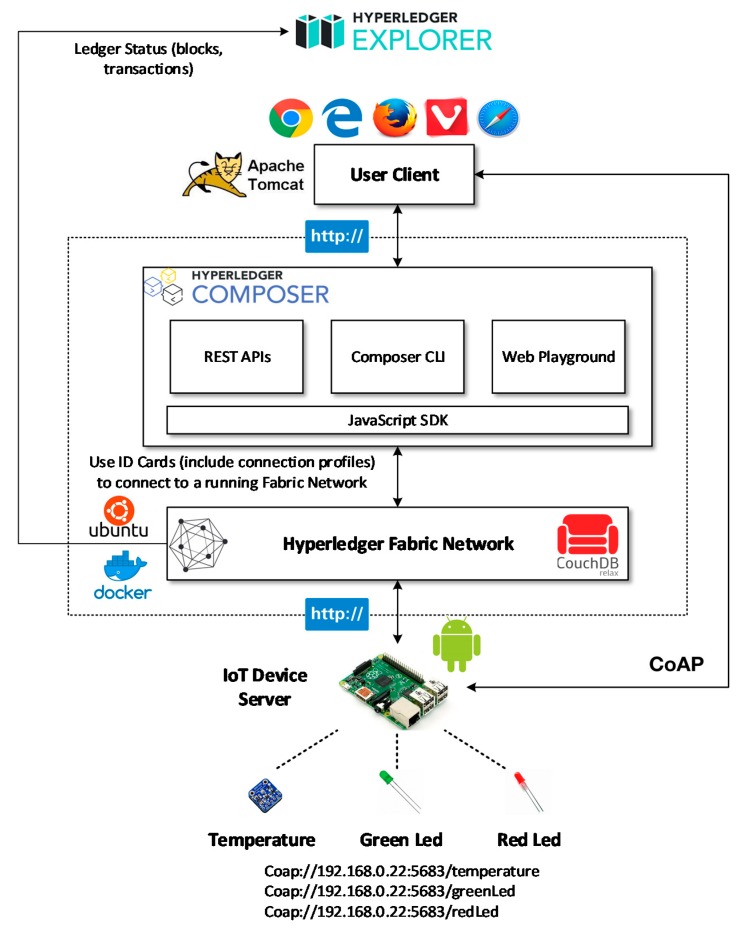
Development solution architecture of the prototype.

In this paper, Hyperledger Composer [35] is used as the development toolset to develop and deploy the prototype blockchain application, which is featured in Figure 6. Hyperledger Composer converts the smart contract for a business network into REST APIs and, at runtime, implements Create, Read, Update, and Delete support to enable transactions to be submitted for processing or retrieval. It supports the existing Hyperledger Fabric blockchain infrastructure and runtime. The Ubuntu virtual machine serves as the hosting environment for docker containers, in which all of the network entities are running as docker images. The JavaScript software development kit (SDK) is used to manage and interact with the Fabric network. Connection profiles are used across Hyperledger Composer to specify how to connect to a Fabric network. They are part of business network cards, which contains IP addresses and ports for the Fabric peers, as well as cryptographic certificates. Couch DB acts as the world state to hold the current states of the ledger. Hyperledger Explorer [36] is a visualization tool used to view ledger status, including blocks, transactions, and other related network information.

The Raspberry Pi is flashed as an android device using Android Things, so the IoT device server residing on the Raspberry Pi is programmed using the Java language. The communication between the IoT device server and the blockchain network happens through REST APIs over HTTP while, for the interaction between the IoT device server and the user client, CoAP is used. One temperature sensor and two LEDs form the physical resources of the device server, and each resource is assigned with a unique URI to be identified by the server. 

The user client is a web-based application developed by various frontend languages such as HTML, CSS, and JavaScript. Apache Tomcat is used as the webserver to serve the web application. The user client visualizes data from the IoT device server and the Fabric network and provides various interfaces to perform operations on the blockchain network. To invoke the smart contract, the user client sends requests to REST APIs by HTTP requests using GET or POST. 

### 4.2. Smart Contract Implementation

**Figure 7:**
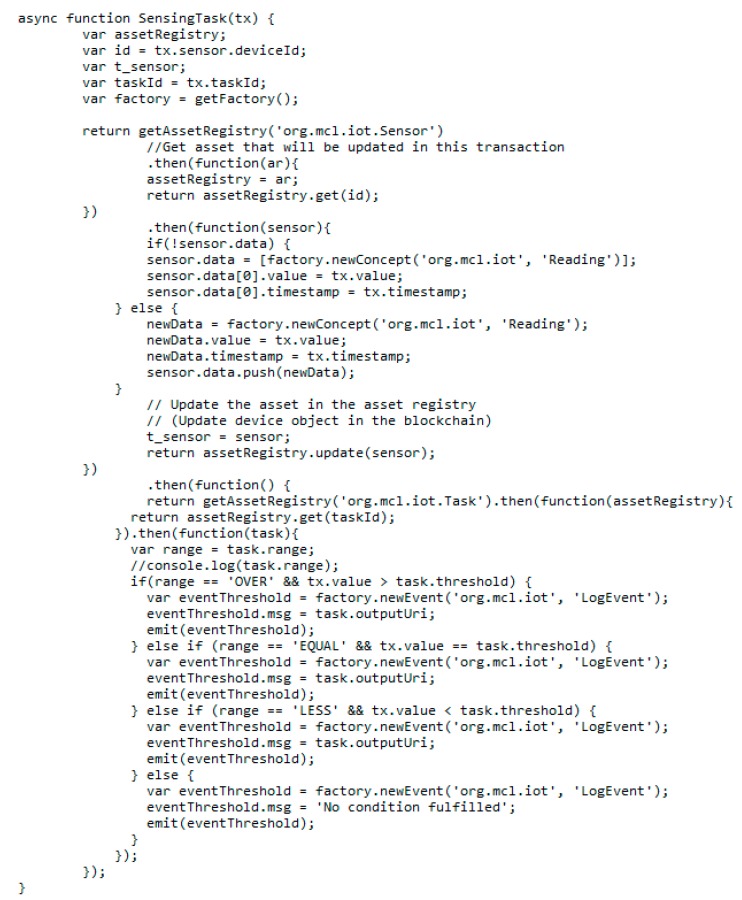
SensingTask transaction processor function.

The smart contract is modeled and packaged as a compressed business network definition, which consists of participants, assets, and transactions. Transaction processor functions define all kinds of operations on participants and assets to either create, update, or delete properties of participants and assets. Figure 7 shows a sample transaction processor function related to the sensing task transaction that is written in JavaScript. This function first updates the corresponding sensor asset by adding a new instance of the sensing data. Afterward, conditional statements are performed according to the threshold defined in the task asset. If the condition is fulfilled, the smart contract will trigger the event to inform the client to allocate the actuating task.

**Figure 8:**
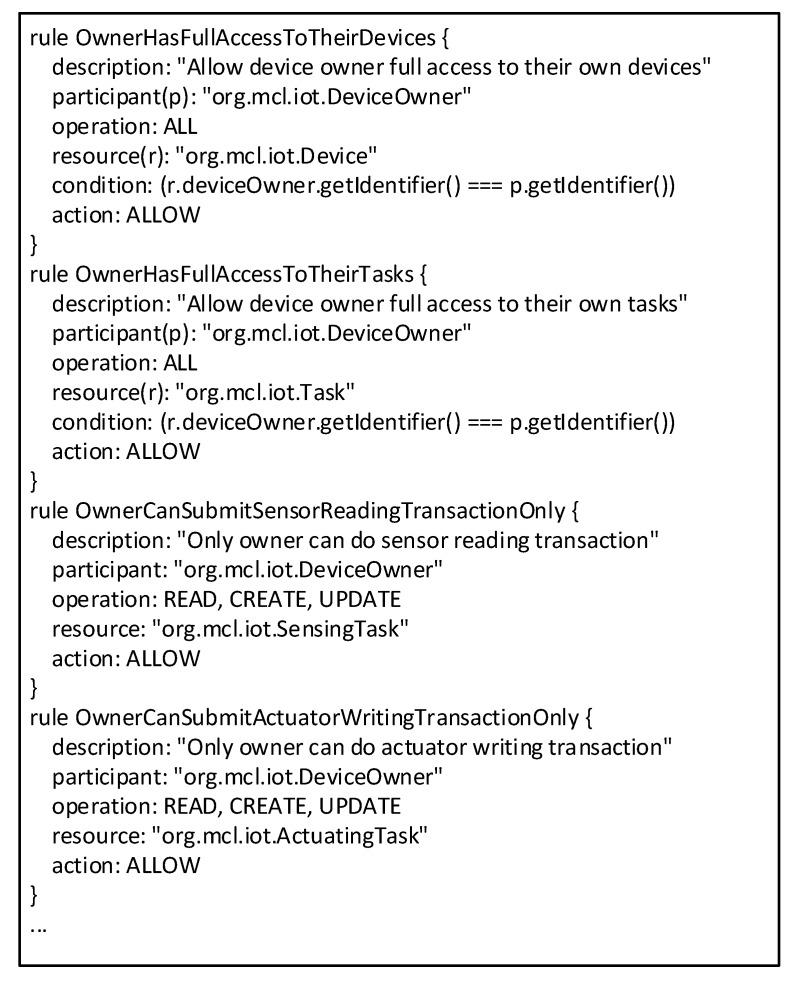
Access control rules in the smart contract.

Access control rules are specified to permit or reject access to resources from specified participants according to the requirements of applications. Figure 8 details some sample rules that are defined for the clinical trial. Four operation types are accessible in the access control rule: READ, CREATE, UPDATE, and DELETE. For example, only the device owner has full operations on devices and tasks, thus preventing access from unauthorized users.

The functions provided by the smart contract are exposed to REST APIs, and a sample list of these APIs is depicted in Table 1. Each API contains a URI, a media type, HTTP verbs (GET, POST, PUT, DELETE), and an action for a given operation. 

## 5. Performance Analysis

**Figure 9:**
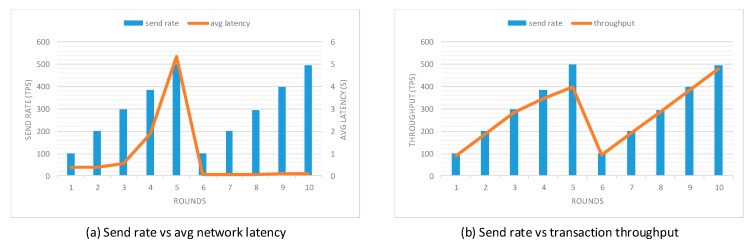
Performance evaluation with the 3-organization-2-peer network model.

**Figure 10:**
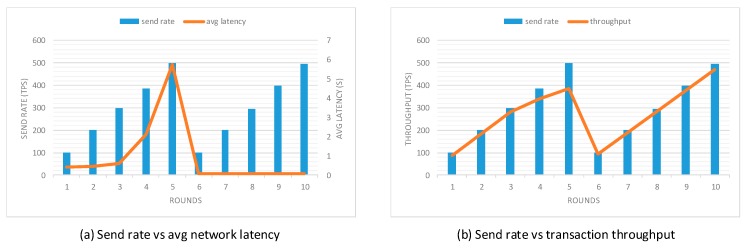
Performance evaluation with the 4-organization-2-peer network model.

**Figure 11:**
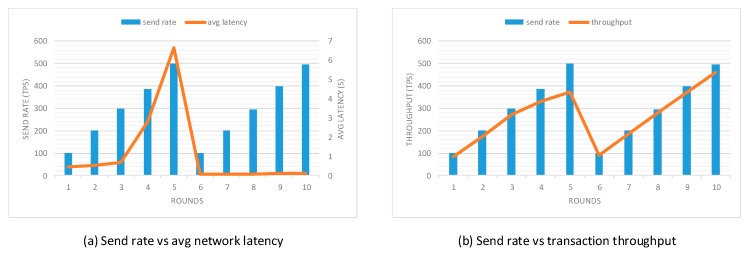
Performance evaluation with the 5-organization-2-peer network model.

To evaluate the performance of our prototype application, an open-source benchmark simulation tool called Hyperledger Caliper [37] was used. We modified the sample network provided by Hyperledger Caliper to simulate the behavior of the proposed prototype application. This experiment was conducted using five local clients and three different network scales, including 3-organization-2-peer, 4-organization-2-peer, and 5-organization-2-peer networks. Each organization contained two peers. Additionally, a single orderer node was used to do transaction ordering and to test functions specified from the smart contract. In this evaluation, two performance indicators were used: network latency and transaction throughput. The network latency measured the amount of time taken for a transaction to be confirmed within the network, including the time from the point that it was submitted but also the time for transaction broadcast and validation as the consensus approach happened. The transaction throughput measured the number of valid transactions that were committed by the blockchain within the allotted time. To accurately evaluate the transaction processing capability of the proposed prototype application, scripts used for the experiment were specified to target two functions, which are task generation and task query transaction, since these two transactions are most frequently invoked by the user client. Ten rounds of tests with a fixed number of transactions were performed with different send rates from 100 tps to 500 tps. The first five rounds performed the task generation transaction and the rest five rounds performed the task query transaction. The experiment was performed in multiple rounds to reduce the probability of errors resulting from system overload and network congestion. Network latency and transaction throughput in different rounds of three sample networks are presented in Figure 9, Figure 10 and Figure 11, respectively. 

Performance evaluation results indicate that with the increase in send rate, the transaction throughput increases linearly as expected until it flattens out at saturation. When the send rate is close to or above the saturation point, the growth rate of transaction throughput decreases significantly. When performing the task generation function, the saturation point in 3-organization-2-peer, 4-organization-2-peer, and 5-organization-2-peer networks are around 347 tps, 339 tps, and 333 tps, respectively. Unlike the task generation transaction, the transaction throughput of task query transaction increases linearly with the increase of send rate. The reason is that task generation transaction requires much more computing power as this function directly modifies the ledger state, while task query transaction only performs read operations on the ledger. Moreover, it is evident from the results that the increase in the number of peers decreases the transaction throughput and increases the network latency of the prototype, which complies with the property of permissioned blockchains.

## 6. Featured Applications and Challenges

This paper presents a prototype blockchain application to justify the usability and feasibility of the proposed solution. The results can be further extended into many other application scenarios, such as home automation, manufacturing, and supply chain management. For example, the significance of this work can bring benefits to the existing shipping industry. Various IoT sensors or tags can be attached to the good that is entrusted to third-party logistics for transport, with trackable record histories, such as location, humidity, and temperature data that are accessible to authorized users. Additionally, the smart contract can monitor the running status of these devices and handle events in terms of specified rules. For example, the smart contract can inform the transport staff if the temperature value exceeds the defined threshold. 

There exist some challenges in this work that need to be discussed and further addressed in future work. One challenge is that the communication between the device and the REST server lacks the authentication that may raise security issues. In the production environment, the REST server should be configured with authentication strategies before devices are allowed to invoke these APIs. Another challenge is that the prototype utilizes the solo ordering service, which features only one single ordering node in the blockchain network. A single ordering node is not fault tolerant since a single point of failure may occur. For that reason, solo implementations cannot be considered for the production environment, but they are the right choices for testing applications and smart contracts since the solo orderer requires much less overhead to get up and to run. In future work, the ordering service will be implemented using crash fault tolerant approaches such as Kafka or Raft, which support multiple ordering nodes. Lastly, the prototype blockchain application is deployed in a local area network to ease the implementation. Considering the requirements in real-world applications, the blockchain infrastructure will be deployed in a cloud environment in future work. 

## 7. Conclusions and Future Research Direction

This paper proposes a reliable task management scheme base on the smart contract to provide runtime verification and allows trustworthy access control to IoT devices in a decentralized manner. If the prerequisites are met, the smart contract would run and enforce the rule as defined. A lightweight solution is proposed that avoids integrating blockchain technology into IoT devices, and alternatively, a RESTful interface that handles requests from devices is set to enable communication between devices and blockchain network. A case study based on Hyperledger Fabric is implemented on top of the proposed architecture, and the evaluation results indicate the efficiency of the system. The prototype application is tested on a different scale of networks, and the evaluation results show a steady transaction processing capability with a low level of resource utilization. 

The future work will refine the case study for evaluating the usability of the proposed solution in practice. First of all, the blockchain infrastructure will be implemented as a flexible blockchain-as-a-service (BaaS) to ease the configuration and management of the blockchain network without caring about the underlying infrastructure. Additionally, crash fault tolerant approaches will be tested to avoid the single point of failure in the orderer node. Moreover, different authentication strategies will be tested to secure the data transmission between the device and the REST server. Furthermore, the blockchain network will be extended to support various IoT devices and tested in more complex scenarios.

## Figures and Tables

**Table 1:** Sample RESTful API endpoints in the business network.

URI	Verb	Media Type	Action
{hostname}/api/DeviceOwner	ALL	Application/json	Manage the device owner
{hostname}/api/Sensor	ALL	Application/json	Manage the sensor
{hostname}/api/Actuator	ALL	Application/json	Manage the actuator
{hostname}/api/Task	ALL	Application/json	Manage the task
{hostname}/api/SensingTaskStatus	ALL	Application/json	Manage the status of the sensing task
{hostname}/api/ActuatingTaskStatus	ALL	Application/json	Manage the status of the actuating task
{hostname}/api/SensingTask	POST	Application/json	Execute the sensing task
{hostname}/api/ActuatingTask	POST	Application/json	Execute the actuating task
{hostname}/api/AllocateSensingTask	POST	Application/json	Create the sensing task status
{hostname}/api/AllocateActuatingTask	POST	Application/json	Create the actuating task status
{hostname}/api/CreateDevice	POST	Application/json	Create a device (sensor or actuator)

## References

[1] Bhattacharjee S., Salimitari M., Chatterjee M., Kwiat K., Kamhoua C. Preserving Data Integrity in IoT Networks Under Opportunistic Data Manipulation. Proceedings of the 3rd Intl Conf on Big Data Intelligence and Computing and Cyber Science and Technology Congress (DASC/PiCom/DataCom/CyberSciTech).

[2] Ahmad S., Hang L., Kim D.-H. (2018). Design and Implementation of Cloud-Centric Configuration Repository for DIY IoT Applications. Sensors.

[3] Al-Fuqaha A., Guizani M., Mohammadi M., Aledhari M., Ayyash M. (2015). Internet of Things: A Survey on Enabling Technologies, Protocols, and Applications. IEEE Commun. Surv. Tutorials.

[4] Taylor R., Baron D., Schmidt D. The world in 2025—Predictions for the next ten years. Proceedings of the 2015 10th International Microsystems, Packaging, Assembly and Circuits Technology Conference (IMPACT); Institute of Electrical and Electronics Engineers (IEEE).

[5] Bertino E., Islam N. (2017). Botnets and Internet of Things Security. Computer.

[6] Zhao Z., Min G., Gao W., Wu Y., Duan H., Ni Q. (2018). Deploying Edge Computing Nodes for Large-Scale IoT: A Diversity Aware Approach. IEEE Internet Things J..

[7] Khan M.A., Salah K. (2018). IoT security: Review, blockchain solutions, and open challenges. Futur. Gener. Comput. Syst..

[8] Zheng Z., Xie S., Dai H., Chen X., Wang H. An Overview of Blockchain Technology: Architecture, Consensus, and Future Trends. Proceedings of the 2017 IEEE International Congress on Big Data (BigData Congress).

[9] Hang, Choi, Kim, Hang L., Choi E., Kim  D.-H. (2019). A Novel EMR Integrity Management Based on a Medical Blockchain Platform in Hospital. Electronics.

[10] Gordon W.J., Catalini C. (2018). Blockchain Technology for Healthcare: Facilitating the Transition to Patient-Driven Interoperability. Comput. Struct. Biotechnol. J..

[11] Dubovitskaya A., Xu Z., Ryu S., Schumacher M., Wang F. Secure and Trustable Electronic Medical Records Sharing using Blockchain. Proceedings of the AMIA Annual Symposium Proceedings.

[12] Hang L., Kim D.-H. (2019). Design and Implementation of an Integrated IoT Blockchain Platform for Sensing Data Integrity. Sensors.

[13] Huh S., Cho S., Kim S. Managing IoT devices using blockchain platform. Proceedings of the 2017 19th International Conference on Advanced Communication Technology (ICACT).

[14] Dorri A., Kanhere S.S., Jurdak R., Gauravaram P. Blockchain for IoT security and privacy: The case study of a smart home. Proceedings of the 2017 IEEE International Conference on Pervasive Computing and Communications Workshops (PerCom Workshops).

[15] Mengelkamp E., Notheisen B., Beer C., Dauer D., Weinhardt C. (2017). A blockchain-based smart grid: Towards sustainable local energy markets. Comput. Sci. Res. Dev..

[16] Guan Z., Si G., Zhang X., Wu L., Guizani N., Du X., Ma Y. (2018). Privacy-Preserving and Efficient Aggregation Based on Blockchain for Power Grid Communications in Smart Communities. IEEE Commun. Mag..

[17] Pop C., Cioara T., Antal M., Anghel I., Salomie I., Bertoncini M. (2018). Blockchain Based Decentralized Management of Demand Response Programs in Smart Energy Grids. Sensors.

[18] Hang L., Kim D.-H. (2019). SLA-Based Sharing Economy Service with Smart Contract for Resource Integrity in the Internet of Things. Appl. Sci..

[19] Huckle S., Bhattacharya R., White M., Beloff N., Huckle S. (2016). Internet of Things, Blockchain and Shared Economy Applications. Procedia Comput. Sci..

[20] Xu L., Shah N., Chen L., Diallo N., Gao Z., Lu Y., Shi W. Enabling the Sharing Economy. Proceedings of the ACM Workshop on Internet of Things (IoT) Security: Issues and Innovations—IoTSec’17.

[21] Cachin C. (2016). Architecture of the hyperledger blockchain fabric. Workshop on Distributed Cryptocurrencies and Consensus Ledgers.

[22] Brewer E. (2000). Towards Robust Distributed Systems.

[23] Incki K., Ari I., Sozer H. Runtime verification of IoT systems using Complex Event Processing. Proceedings of the 2017 IEEE 14th International Conference on Networking, Sensing and Control (ICNSC); Institute of Electrical and Electronics Engineers (IEEE).

[24] Incki K., Ari I. (2018). A Novel Runtime Verification Solution for IoT Systems. IEEE Access.

[25] Aktas M.S., Astekin M. (2017). Provenance aware run-time verification of things for self-healing Internet of Things applications. Concurr. Comput. Pr. Exp..

[26] Incki K., Ari I. (2018). Democratization of runtime verification for internet of things. Comput. Electr. Eng..

[27] Yu K., Chen Z., Dong W. A Predictive Runtime Verification Framework for Cyber-Physical Systems. Proceedings of the 2014 IEEE Eighth International Conference on Software Security and Reliability-Companion.

[28] Atlam H.F., Alenezi A., Alassafi M.O., Wills G. (2018). Blockchain with Internet of Things: Benefits, Challenges, and Future Directions. Int. J. Intell. Syst. Appl..

[29] Dorri A., Kanhere S.S., Jurdak R. Towards an Optimized BlockChain for IoT. Proceedings of the Second International Conference on Internet-of-Things Design and Implementation—IoTDI’17.

[30] Novo O. (2018). Blockchain Meets IoT: An Architecture for Scalable Access Management in IoT. IEEE Internet Things J..

[31] Cha S.-C., Chen J.-F., Su C., Yeh K.-H. (2018). A Blockchain Connected Gateway for BLE-Based Devices in the Internet of Things. IEEE Access.

[32] Xiong Z., Zhang Y., Niyato D., Wang P., Han Z. (2018). When mobile blockchain meets edge computing. IEEE Commun. Mag..

[33] Guo S., Hu X., Guo S., Qiu X., Qi F. (2019). Blockchain meets edge computing: A distributed and trusted authentication system. IEEE Trans. Ind. Inform..

[34] Yang R., Yu F.R., Si P., Yang Z., Zhang Y. (2019). Integrated Blockchain and Edge Computing Systems: A Survey, Some Research Issues and Challenges. IEEE Commun. Surv. Tutor..

[35] Gaur N., Desrosiers L., Ramakrishna V., Novotny P., Baset S.A., O’Dowd A. (2018). Hands-On Blockchain with Hyperledger: Building Decentralized Applications with Hyperledger Fabric and Composer.

[36] Hyperledger Explorer—A Useful Tool to View Blocks Transactions on Hyperledger Fabric. https://www.hyperledger.org/projects/explorer.

[37] Hyperledger Caliper—A Blockchain Benmark Tool. https://www.hyperledger.org/projects/caliper.

